# The Zinc Transporter *Zip5* (*Slc39a5*) Regulates Intestinal Zinc Excretion and Protects the Pancreas against Zinc Toxicity

**DOI:** 10.1371/journal.pone.0082149

**Published:** 2013-11-26

**Authors:** Jim Geiser, Robert C. De Lisle, Glen K. Andrews

**Affiliations:** 1 Department of Biochemistry and Molecular Biology, University of Kansas Medical Center, Kansas City, Kansas, United States of America; 2 Department of Anatomy and Cell Biology, University of Kansas Medical Center, Kansas City, Kansas, United States of America; Centro Nacional de Investigaciones Oncológicas (CNIO), Spain

## Abstract

**Background:**

ZIP5 localizes to the baso-lateral membranes of intestinal enterocytes and pancreatic acinar cells and is internalized and degraded coordinately in these cell-types during periods of dietary zinc deficiency. These cell-types are thought to control zinc excretion from the body. The baso-lateral localization and zinc-regulation of ZIP5 in these cells are unique among the 14 members of the Slc39a family and suggest that ZIP5 plays a role in zinc excretion.

**Methods/Principal Findings:**

We created mice with floxed *Zip5* genes and deleted this gene in the entire mouse or specifically in enterocytes or acinar cells and then examined the effects on zinc homeostasis. We found that ZIP5 is not essential for growth and viability but total knockout of ZIP5 led to increased zinc in the liver in mice fed a zinc-adequate (ZnA) diet but impaired accumulation of pancreatic zinc in mice fed a zinc-excess (ZnE) diet. Loss-of-function of enterocyte ZIP5, in contrast, led to increased pancreatic zinc in mice fed a ZnA diet and increased abundance of intestinal *Zip4* mRNA. Finally, loss-of-function of acinar cell ZIP5 modestly reduced pancreatic zinc in mice fed a ZnA diet but did not impair zinc uptake as measured by the rapid accumulation of ^67^zinc. Retention of pancreatic ^67^zinc was impaired in these mice but the absence of pancreatic ZIP5 sensitized them to zinc-induced pancreatitis and exacerbated the formation of large cytoplasmic vacuoles containing secretory protein in acinar cells.

**Conclusions:**

These studies demonstrate that ZIP5 participates in the control of zinc excretion in mice. Specifically, they reveal a paramount function of intestinal ZIP5 in zinc excretion but suggest a role for pancreatic ZIP5 in zinc accumulation/retention in acinar cells. ZIP5 functions in acinar cells to protect against zinc-induced acute pancreatitis and attenuate the process of zymophagy. This suggests that it may play a role in autophagy.

## Introduction

Zinc homeostasis is tightly controlled which reflects the essential functions of this metal in a vast array of proteins including enzymes, transcription factors, cell surface receptors and proteins involved in signalling cascades [1], [2]. Ultimately when zinc is deficient, cell division, growth and viability are impaired. Control of zinc homeostasis is exerted predominately by three families of proteins [3]–[6]. The most abundant and ubiquitously expressed members of the cysteine-rich metallothionein family (MT-I and II in mice) are induced by zinc and function as intracellular zinc buffers which provide a biologically available pool of zinc. Over-expression of these genes in mice provides protection against dietary zinc deficiency whereas loss-of -function renders mice more sensitive to zinc deficiency [7], [8]. Uptake and efflux of zinc involve two diverse families of zinc transporters. Members of the *Slc39a* or *Zip* family (14 known genes) are thought to transport zinc into the cytoplasm of cells, either from the extracellular milieu or from the vesicular compartment [5]. Some of these family members may also transport other essential metals such as iron or cadmium, and many display cell-specific patterns of expression and regulation [9]–[12]. Members of the Slc30a or *ZnT* family (10 known genes) are generally thought to efflux zinc out of the cytosol and into the extracellular milieu or into the vesicular compartment [3]. As noted above, family members may also play an important role in the transport of other metals such as manganese [13] and many display cell specific patterns of expression [14]. The complexity of the protein families involved in zinc homeostasis clearly reflects the diverse functions of this essential metal.

Recent genetic studies have begun to reveal physiological roles of many of the members of these two zinc transporter families. Among the 14 members of the *Zip* gene family, 7 have been mutated in mice and the physiological consequences examined. Our studies of *Zips1, 2 and 3*, members of a highly conserved subfamily, revealed that these transporters are not essential. However, they each play unique tissue-specific roles during zinc deficiency [12], [15]. In contrast, we demonstrated that *Zip4* is an essential gene in mice and expression of this gene specifically in the intestinal epithelium or yolk sac endoderm mediates the acquisition of dietary zinc in newborn and adult mice or by the early embryo, respectively [16], [17]. Loss-of-function of this gene leads to wasting unless these mice are maintained on high levels of zinc [17]. The *Zip4* gene is mutated in humans with acrodermatitis enteropathica, a potentially lethal zinc deficiency disease [18], [19]. Studies of mice expressing a *Zip8* hypomorphic allele revealed that active expression of this gene is essential during late fetal and early postnatal life and is important for multi-organ development [20]. This gene has also been shown to increase sensitivity to cadmium toxicity [21]. Other recent studies found that *Zip13* is not essential for viability, but deletion of this gene results in impaired connective tissue development in mice [22]. This results in changes in bone, teeth and connective tissue similar to that noted in humans with Ehlers-Danlos syndrome, some of whom have mutations in this gene [22]. Finally, mice lacking *Zip14* exhibit growth retardation with impaired gluconeogenesis and reduced hepatocyte proliferation during liver regeneration [23], [24].

In the current study we probed the physiological roles of *Zip5* (*Slc39a5)* in zinc homeostasis. This zinc transporter is particularly interesting because it localizes to the baso-lateral cell membrane and is abundant specifically in intestinal enterocytes, pancreatic acinar cells and embryonic visceral endoderm cells [25]. These cell types play key roles in mammalian zinc homeostasis. ZIP5 regulation also appears to be unique in that this protein is internalized and degraded coordinately in each of these cell-types during periods of dietary zinc deficiency [25], [26]. Translation of the *Zip5* mRNA is stalled during zinc deficiency in a mechanism which involves a conserved 3′-untranslated region that is predicted to form a stable stem-loop structure and to interact with specific microRNAs [26], [27]. Given its unique zinc regulation, cellular localization and active expression in organs involved in the control of zinc homeostasis in mammals, we created mice with floxed Zip5 genes and examined mice in which this gene was disabled specifically in enterocytes or acinar cells or disabled in all cells. We found that this gene is not essential for growth and viability of mice. However, our studies revealed a novel role for enterocyte ZIP5 in zinc excretion, and a role for pancreatic acinar cell ZIP5 in protection against zinc toxicity. Loss-of-function of enterocyte ZIP5 led to increased accumulation of pancreatic zinc whereas loss-of-function of acinar cell ZIP5 exacerbated the formation of large cytoplasmic granules containing secretory protein and acinar cell atrophy during zinc-induced acute-pancreatitis. These findings suggest a role for acinar cell ZIP5 and/or zinc in a process termed zymophagy, the selective autophagy of secretory granules [28], [29].

## Results

### Knocking out the *Zip5* gene in every cell, in the intestinal epithelium, or in pancreatic acinar cells

**Figure 1:**
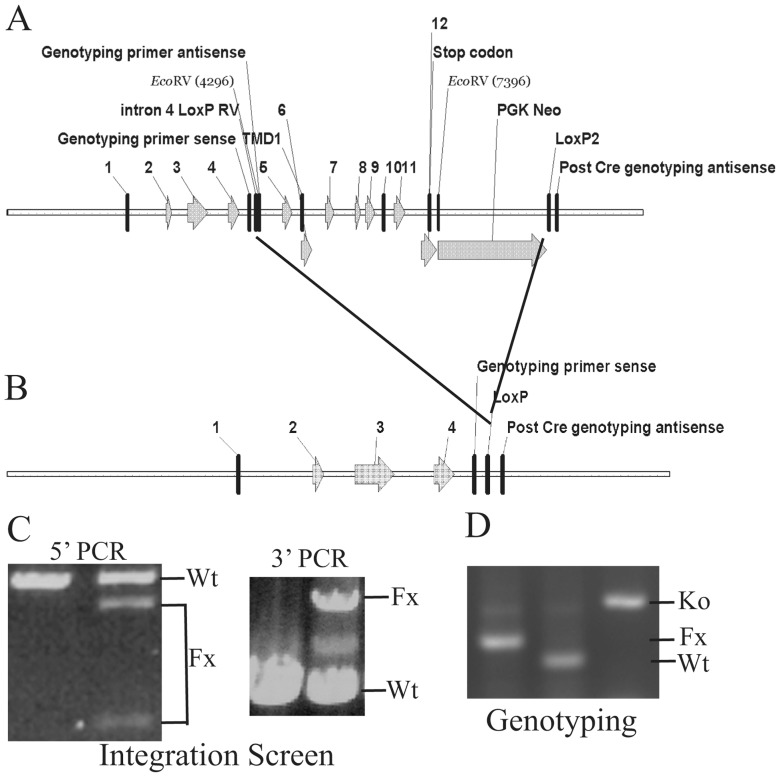
Structures of the pre- and post-Cre floxed mouse *Zip5* gene and integration and genotyping screen designs. (**A**) The mouse *Zip5* gene was captured using gap-repair and manipulated using galK recombineering. Exons (**1–12**) and the exon encoding transmembrane domain 1 (**TMD1**) are indicated, as are the positions of ***LoxP*** sites (intron 4 and downstream of *PGK Neo*), the *PGK-neomycin* (***PGK Neo***) cassette and the locations of primers used for genotyping. The *LoxP* site in intron 4 is flanked by an *EcoRV* restriction enzyme cleavage site. (**B**) The structure of the *Zip5* gene after Cre recombination is shown. Recombination eliminates the transmembrane domain of ZIP5. (**C**) The floxed *Zip5* gene was targeted into E14 ES cells and properly targeted ES cells were identified by long range PCR using flanking and internal primers. PCR products from the wild-type (***Wt***) and floxed (***Fx***) alleles are indicated. *EcoRV* cleavage was used to differentiate between the floxed and wild-type alleles in the **5′ PCR** screen whereas the **3′ PCR** screen yielded the predicted larger product from the wild-type allele. Targeted ES cells were used to generate mice homozygous for the floxed *Zip5* allele. (**D**) Mice were genotyped by PCR amplification of the intron 4 region containing the *LoxP* site. The PCR product from homozygous *Zip5* floxed mice before Cre-induced recombination is shown in the **left lane** while that from control mice is shown in the **center lane** and that from *Zip5*-knockout mice (**Ko**) is shown in the **right lane**. For the intestine- and pancreas-specific knockout mice, detection of *Zip5* mRNA and/or protein was employed to monitor the efficacy of recombination.

A mouse *Zip5* gene targeting construct was created using BAC recombineering. A *LoxP* site flanked by an *EcoRV* restriction site was inserted into intron 4, and a second *LoxP* site was inserted downstream from a *PGK-Neomycin* cassette placed after the last exon in the *Zip5* gene (Fig. 1A). Recombination of this construct results in the removal of the entire transmembrane domain of ZIP5 leaving only exons 1–4 intact (Fig. 1B). This construct was targeted in E14 embryonic stem cells and homologous integration of the floxed *Zip5* gene was identified using long-range PCR and primers outside of the engineered targeting construct coupled with overlapping internal primers (Fig. 1C). The 5′ integration screen (Fig. 1C) amplified a 6.22 kb product from the wild-type and the floxed alleles and cleavage of the floxed allele with *EcoRV* yielded the predicted 4.5 and 1.7 kb restriction fragments indicative of homologous recombination of the floxed allele. This was confirmed by the 3′ integration screen which yielded a 3.39 kb PCR product from the floxed allele and a 5.26 kb PCR product from the wild-type allele. Targeted E14 ES cells were used to create chimeric mice by blastocyst injection and agouti offspring from the chimeric mice were genotyped using primers which flank the *LoxP-EcoRV* insertion site in intron 4 (Fig. 1D). Genotyping PCR yielded a 197 bp product from the floxed allele which could be cut by *EcoRV*, a 157 bp product from the wild-type allele, and a 275 bp product from the recombined allele after CRE excision (Fig. 1D).

### The mouse *Zip5* gene is not essential but loss-of-function leads to changes in zinc accumulation in the liver, intestine and pancreas

Total knockout of the *Zip5* gene was accomplished by crossing *Zip5^Fx/Fx^* mice with mice that express Cre under control of the ubiquitously expressed EIIa promoter. Mice showing complete recombination of the *Zip5^Fx/Fx^* alleles were selected to develop a working colony and age matched *Zip5^Fx/Fx^* mice were used as controls. *Zip5*-knockout mice displayed normal fecundity (6.6±1.8 KO vs 7.0±1.9 control pups per litter) and had no overt phenotype up to 1 year of age when they were no longer active breeders and were euthanized. Thus, the mouse *Zip5* gene is not essential.

**Figure 2:**
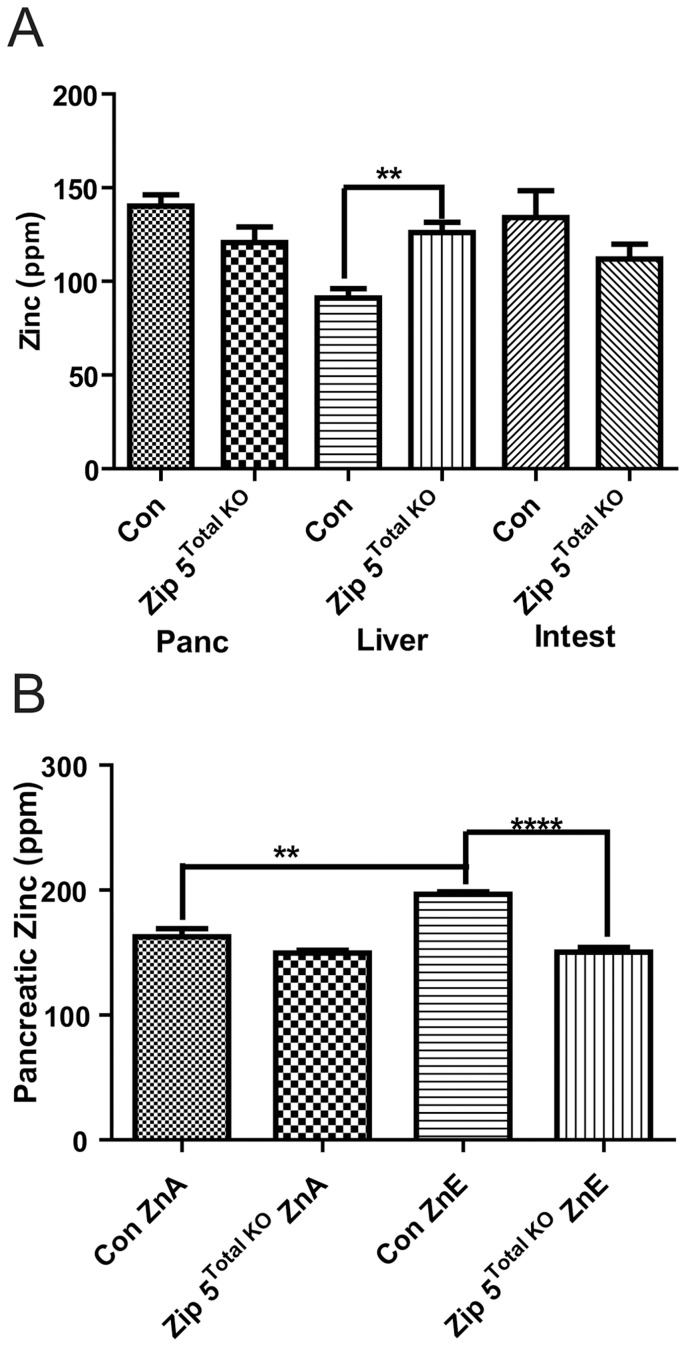
Total knockout of the *Zip5* gene results in increased hepatic zinc and modestly attenuates pancreatic zinc accumulation. Mice with homozygous floxed *Zip5* genes were crossed with mice that ubiquitously express CRE recombinase under control of the *EIIa* promoter. Offspring with complete recombination of the *Zip5* gene in tail DNA were identified and crossed to create a colony of *Zip5*- knockout mice (*Zip5 ^Total KO^*). *Zip5 ^Fx/Fx^* mice served as controls (Con). (A) Mice (n = 4 or 5) were maintained on normal diet (ZnA) diet and pancreas (Panc), liver and intestine (Intest) were harvested two weeks after weaning and from age matched *Zip5 ^Total KO^* and control and tissue elements were quantified using ICP-MS and are expressed as ppm/dry weight. Zinc in the liver was the only element which differed significantly between the *Zip5 ^Total KO^* and control mice (P = 0.0032). (B) Intestine, pancreas and liver were harvested from control and *Zip5 ^Total KO^* mice fed normal chow (ZnA) or normal chow plus 250 ppm zinc in the drinking water (ZnE) for 8 days and tissue elements were quantified using ICP-MS. Only pancreatic zinc is shown since no other significant changes in any other element were found (**; P<0.005: ****; P<0.0001).

However, elemental analyses of major organs (liver, pancreas and intestine) involved in zinc homeostasis revealed a significant increase (∼38%) in liver zinc in the knockout mice and a trend toward lower levels of zinc in the intestine and pancreas in mice fed a zinc adequate diet (ZnA), although the latter did not reach statistical significance (Fig. 2A). There were no other significant differences in the multiple essential metals and other elements measured using inductively-coupled plasma mass spectrometry (ICP-MS). Mice were then challenged chronically by providing drinking water containing excess zinc (250 ppm zinc; ZnE) for 8 days. Tissues were analyzed by ICP-MS (Fig. 2B). Whereas control mice provided with drinking water containing excess zinc (ZnE) for 8 days accumulated zinc in the pancreas, these knockout mice did not (Fig. 2B). This was the only significant difference noted in all elements examined. The above results show that ZIP5 functions to modulate zinc homeostasis and suggest that the absence of this zinc transporter leads to an increase in the burden of zinc causing accumulation in the liver instead of accumulation in the pancreas which normally occurs (see below).

### Loss-of-function of the intestine *Zip5* gene leads to increased accumulation of zinc in the pancreas

The above results are consistent with ZIP5 playing a role, albeit subtle, specifically in zinc homeostasis, but total knockout does not address tissue-specific roles for this zinc transporter. To explore this possibility we examined the effects of conditionally deleting this gene in the intestine and pancreas, the organs which most actively express the *Zip5* gene. ZIP5 protein accumulates on the basolateral surfaces of enterocytes and acinar cells, cell-types which are thought to play key roles in mammalian zinc homeostasis.

**Figure 3:**
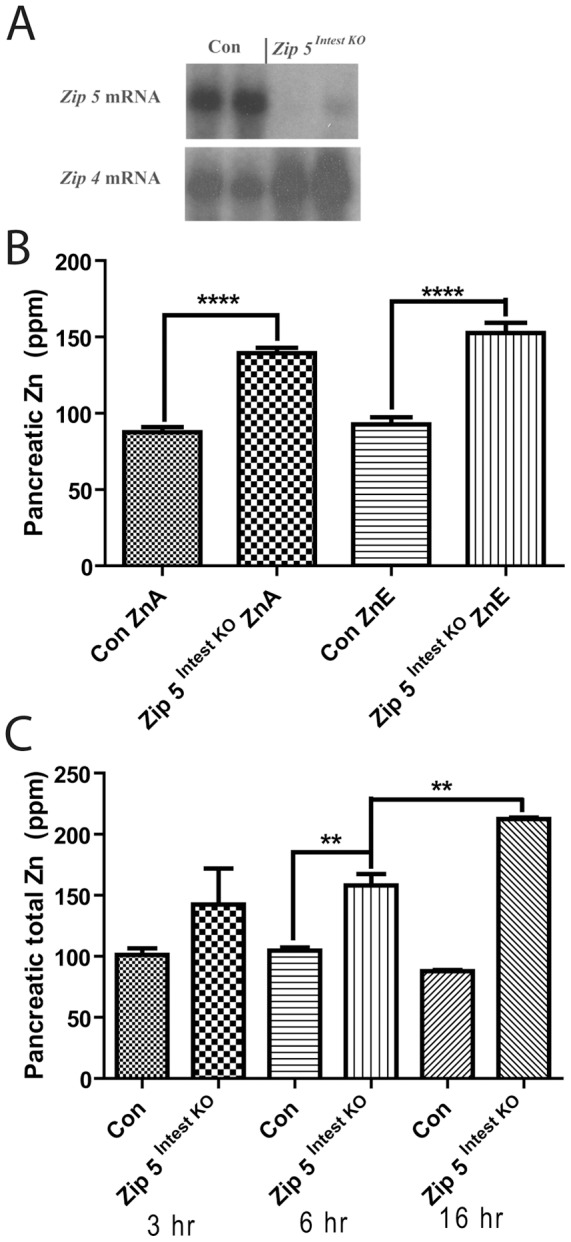
Loss of function of the intestine *Zip5* gene results in the accumulation of zinc in the pancreas. Newly weaned mice were injected with tamoxifen to induce *Zip5* recombination in the intestine by activation of Cre-ERT2 expressed from a *vil-Cre-ERT2* transgene. (**A**) Northern blot detection of *Zip5* and *Zip4* mRNAs in the small intestine of control (**Con**) littermates and intestine-specific *Zip5*-knockout (***Zip5 ^Intest KO^***) mice. Results from 2 mice per group are shown but 5 mice per group were analyzed with identical results. (**B**) Intestine, pancreas and liver were harvested from control and *Zip5^Intest KO^* mice fed normal chow (**ZnA**) or normal chow plus 250 ppm zinc in the drinking water (**ZnE**) for 8 days after knocking out the *Zip5* gene in the intestine. Tissue elements were quantified using ICP-MS and are expressed as ppm/dry weight of tissue. Only zinc levels in the pancreas are shown because this was the only change detected (n = 5: ****; P<0.0001). (**C**) Control and *Zip5^Intest KO^* mice (n = 3) were given a gavage containing 30 ppm zinc and zinc levels in the pancreas, liver and intestine were monitored using ICP-MS at the indicated times (**3 to 16 h**) thereafter. No significant change in zinc or any other element was noted in the liver and intestine. Only zinc increased in the pancreas of the *Zip5^Intest KO^* mice. (**; P<0.006).

To enable tissue-specific recombination of the floxed *Zip5* gene specifically in the intestinal epithelium, newly weaned *Zip5^Fx/Fx^* mice expressing an estrogen receptor-Cre recombinase fusion protein under control of the *villin* promoter [30] and *Zip5^Fx/Fx^* littermates that do not expresses the transgene were injected with tamoxifen to drive the Cre-ERT2 fusion protein to the nucleus and initiate recombination of the floxed gene. Northern blot analysis of RNA from the small intestine of *Zip5 ^Intest KO^* mice revealed a dramatic reduction in *Zip5* mRNA and an increase in the abundance of Zip4 mRNA (Fig. 3A). Efficacy of the knockout was examined in multiple mice and was estimated to be >90%.

Elemental analysis of the pancreas revealed a ∼60% increase in pancreatic zinc in the *Zip5 ^Intest KO^* mice fed a zinc adequate (ZnA) diet (Fig. 3B) but no change in liver or intestine zinc (data not shown). This difference between control and *Zip5 ^Intest KO^* mice was exacerbated in mice provided excess zinc in the drinking water for 8 days. However, in this experiment neither the control nor *Zip5 ^Intest KO^* mice showed a statistically significant increase in pancreatic zinc in response to chronic exposure to excess dietary zinc. In contrast, figures 2B and 5A show that excess dietary zinc normally leads to significant increases in zinc in the pancreas of control mice. The reason for this discrepancy is unclear but the results in figure 3B clearly demonstrate that the *Zip5^Intest KO^* pancreas contains much higher levels of zinc than does that of control mice. After acute exposure to excess zinc by gavage, *Zip5 ^Intest KO^* mice accumulated significantly more pancreatic zinc than did control mice (Fig. 3C) confirming that loss-of-function of intestine ZIP5 causes increased accumulation of pancreatic zinc. There were no significant differences between the *Zip5 ^Intest KO^* and control mice in the other organs or other elements examined in these experiments.

**Figure 4:**
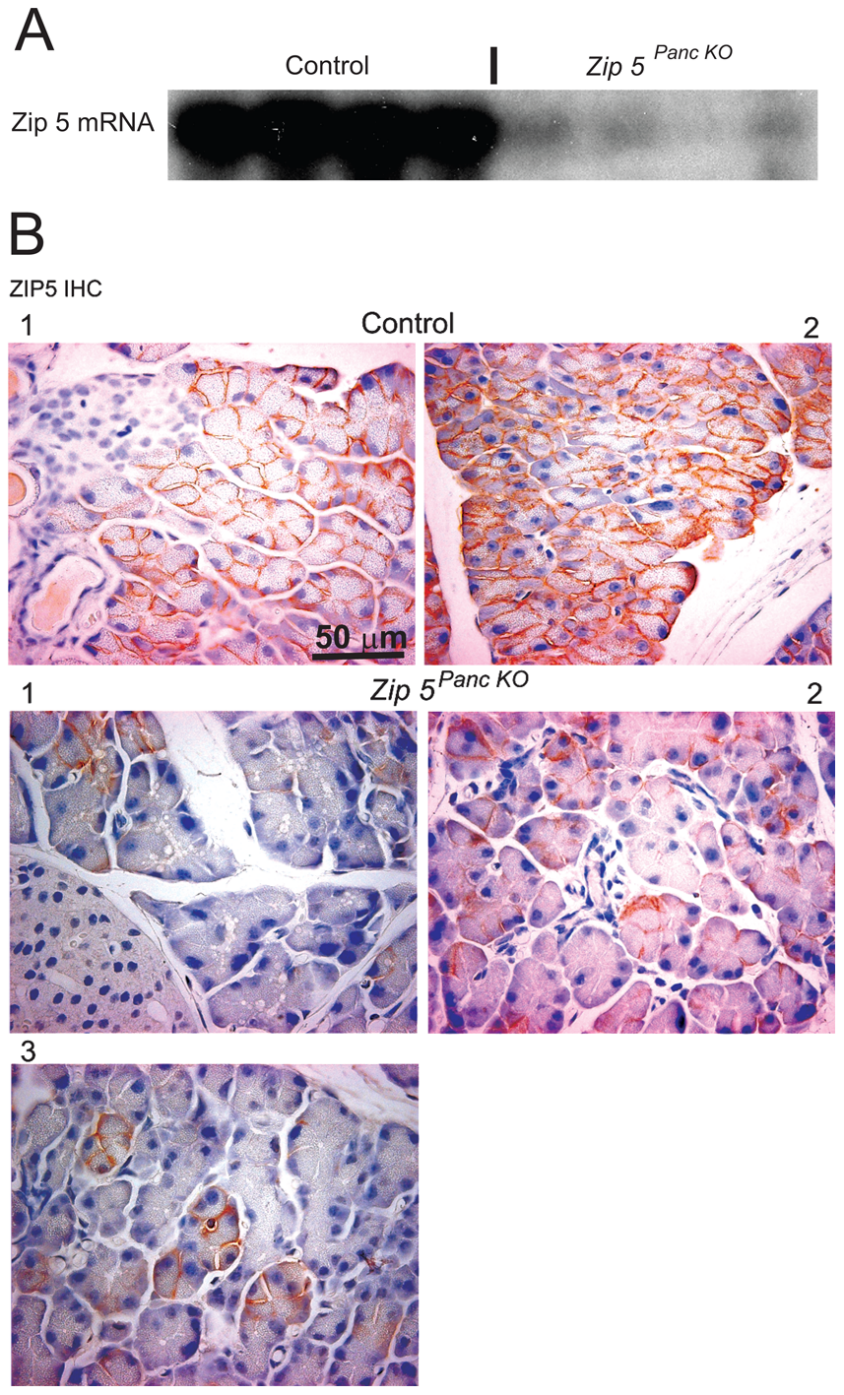
Knockout of the pancreas acinar cell *Zip5* gene is mosaic. Newly weaned mice were injected with tamoxifen to induce *Zip5* recombination in the pancreas by activation of Cre-ERT2 expressed from an *Ela-Cre-ERT2* transgene. (**A**) Northern blot detection of *Zip5* mRNA in the pancreas of control (**Control**) littermates and pancreas-specific *Zip5*-knockout (***Zip 5 ^Panc KO^***) mice killed 5 days after the last tamoxifen injection. (**B**) Detection of ZIP5 protein using immunohistochemistry (**ZIP5 IHC**). Paraffin sections of pancreas were incubated with an anti-ZIP5 peptide antibody and specific binding was detected as a dark brown precipitate on the baso-lateral surfaces of acinar cells. Sections from 2 control mice and 3 different knockout mice are shown at 200× magnification. Quantification of the number of ZIP5 immuno-positive and negative cells per field of view in multiple sections from several mice indicated an efficiency of the knockout of ∼70 to ∼90%. In Part B number 1 knockout, the percentage of remaining positive cells was 8% (6/80) indicating a 92% efficacy of the knockout. In part B number 2 knockout, the percentage of remaining positive cells 32% (39/120) indicating an ∼68% efficacy of the knockout. **I**; indicates Islets of Langerhans: **D**; indicates a pancreatic duct.

The above results reveal cross-talk between the intestine and pancreas with regard to zinc homeostasis and are consistent with the concept that ZIP5 plays a role in zinc excretion from the intestine by the uptake of zinc from the blood into the intestine.

### Loss-of-function of the pancreas *Zip 5* gene sensitizes mice to zinc-induced pancreatitis and reduces the accumulation but not the uptake of pancreatic zinc

To enable tissue-specific recombination of the floxed *Zip5* gene in pancreatic acinar cells, newly weaned *Zip5* ^Fx/Fx^ mice that express Cre-ERT2 recombinase under control of the *elastase* promoter [31] and *Zip5^Fx/Fx^* littermates were injected with tamoxifen to initiate recombination of the floxed gene. Northern blot analysis of pancreatic RNA from *Zip5 ^Panc KO^* mice revealed that *Zip5* mRNA was dramatically reduced but remained detectable in many of the samples (Fig. 4A). Detection of ZIP5 by immunohistochemistry (IHC) in sections of the pancreas revealed that the knockout was mosaic (Fig. 4B). Quantification of the extent of the knockout by counting the relative number of positive cells per field of view indicated an efficacy of 70 to 90%.

**Figure 5:**
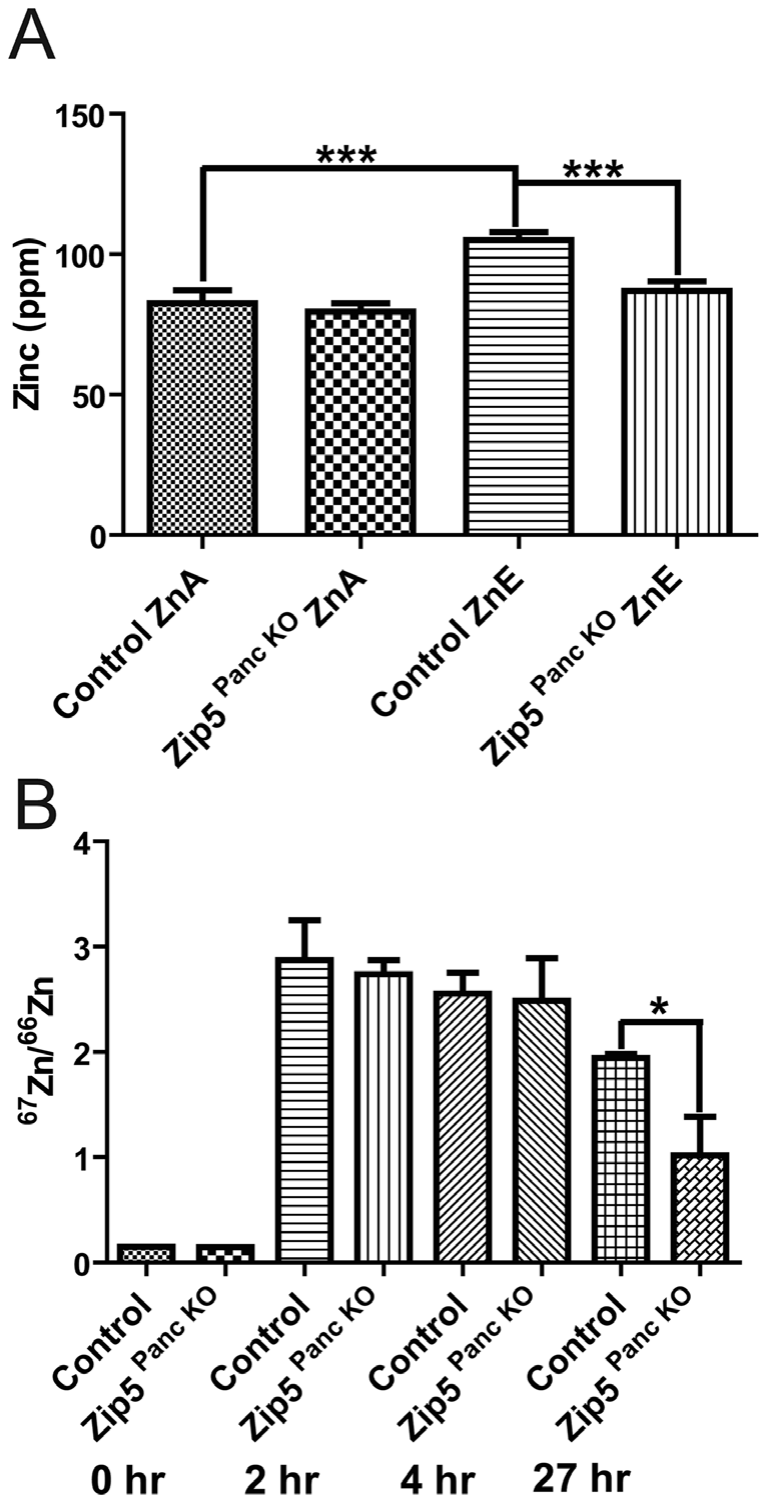
Knockdown of the pancreas acinar cell *Zip5* gene has no effect on the rapid accumulation of zinc but apparently impairs zinc retention in the pancreas. (A) Control littermates and pancreas-specific *Zip5*-knockout (*Zip5 ^Panc KO^*) mice (n = 4– 5) were killed 8 days after the last tamoxifen injection. Intestine, pancreas and liver were harvested from mice fed normal chow (ZnA) or normal chow plus 250 ppm zinc in the drinking water (ZnE) during those 8 days and pancreatic zinc was quantified using ICP-MS and is expressed as ppm/dry weight of tissue. (B) Control and *Zip5 ^Panc KO^* mice were injected I.P. with 6.25 mg ^67^Zn/kg body weight and the pancreas was harvested at 0 h, 2 h and 4 hr after the injection or (C) 27 hr after the injection. The ratio of ^67^Zn/^66^Zn was measured by ICP-MS. The natural ratio of these stable isotopes is 0.146. Only pancreatic zinc is shown since no other significant changes in any other element were found (*; P<0.019; ***; P<0.0007).

Elemental analysis of intestine, liver and pancreas from *Zip5 ^Panc KO^* and control mice revealed a decrease in liver iron (Fig.S1A). Although the amount of pancreatic zinc in control versus *Zip5 ^Panc KO^* mice fed the ZnA diet did not reach statistical significance in the data shown (Fig. 5A), increasing the number of animals in the assay revealed a small but statistically significant decrease in pancreatic zinc in the knockout mice relative to the controls when fed a ZnA diet (Fig. S1B). There were no other statistically significant changes in the abundance of the other elements examined in these organs and no overt phenotypic changes were noted in *Zip5 ^Panc KO^* mice fed a ZnA diet. However, *Zip5 ^Panc KO^* mice provided with zinc in the drinking water for 8 days failed to accumulate significantly more zinc in the pancreas, whereas control littermates accumulated pancreatic zinc under these experimental conditions (Fig. 5A). These results suggest that pancreatic ZIP5 might play a role in the retention of pancreatic zinc.

To further examine that possibility we studied the accumulation and retention of ^67^Zn, a stable isotope of zinc, in these mice. In initial experiments *Zip5 ^Panc KO^* mice and *Zip5 ^Intest KO^* mice were given an oral gavage containing 250 or 500 ppm ^67^Zn and the accumulation of ^67^Zn relative to ^66^Zn was monitored 24 hr later (Fig. S2). The natural ratio of ^67^Zn/^66^Zn is 0.146. In these initial experiments, the data showed a trend toward reduced accumulation of pancreatic zinc in the *Zip5 ^Panc KO^* mice, but variability in the data was high and the results were not statistically significant. In subsequent experiments, mice were given an intraperitoneal (I.P.) injection of ^67^Zn (6.25 mg/kg body weight) and pancreatic accumulation of this stable isotope was monitored (Fig. 5B & C). The results showed that both control and *Zip5 ^Panc KO^* mice accumulated similar amounts of pancreatic ^67^Zn at 2 and 4 hr after the injection (Fig. 5B). Thus, ZIP5 is not essential for the acute uptake of zinc in the pancreas under these conditions. In contrast, by 27 hr after the injection of ^67^Zn (Fig. 5B), the control mice had retained significantly more pancreatic ^67^Zn than had the *Zip5 ^Panc KO^* mice (13.2 fold increase vs 7.07 fold increase over control in ^67^Zn, respectively). These results are consistent with the concept that ZIP5 might function in the pancreas to aid in the retention of zinc but not in the uptake of zinc.

The pancreas is sensitive to zinc-induced pancreatitis [32]. Therefore we investigated the effects of knocking out the pancreatic *Zip5* gene on the development of pancreatitis in response to an I.P. injection of zinc. In an initial experiment, mice were injected with 6.25 mg zinc/kg body weight and 24 hr later the pancreas was harvested, fixed and sectioned. Sections were examined blindly by a pathologist (Table 1). The results revealed that the *Zip5 ^Panc KO^* mice developed a more severe peri-pancreatic inflammation (8/9 of the mice shown in Table 1), as judged by the increased presence of immune cells around the pancreas, than did control mice, but these mice showed no overt signs of toxicity. In addition, remarkably large cytoplasmic vacuoles were noted in pancreatic acinar cells in the majority of the *Zip5 ^Panc KO^* mice treated with zinc (6/9 of the mice shown in Table 1) but were very rare or absent in control mice treated with zinc and absent in untreated control and *Zip5 ^Panc KO^* mice. These vacuoles were often much larger than nuclei. Acinar cell atrophy was present in 2 of nine *Zip5 ^Panc KO^* mice but not in any of the control mice after this zinc treatment. These results suggest that pancreatic injury in response to injected zinc may have also contributed to the diminished retention of pancreatic zinc in the *Zip5 ^Panc KO^* mice (Fig. 5B) and also reveal that pancreatic ZIP5 plays a role in protecting the pancreas against zinc toxicity.

**Table 1:** Zinc-induced pancreatic pathology in control and *Zip5 ^Panc KO^* mice.

Genotype	Large Cytoplasmic Vacuoles	Peripancreatic Inflammation
***Zip5^panc KO^***	No	severe
	Yes	moderate
	Yes	severe
	Yes (prominent)	severe
	Yes (prominent)	severe
	Yes (prominent)	severe
	Yes	severe
	No (atrophy of acinar cells)	severe
	No (atrophy of acinar cells)	severe
**Control**	No	mild to moderate
	No	moderate
	No	moderate
	No	moderate

Newly weaned mice were injected with tamoxifen to induce *Zip5* recombination in pancreatic acinar cells by activation of Cre:ERT2 expressed from an *ela-Cre-ERT2* transgene. Two weeks after the last tamoxifen injection, control (**Control**) littermates and pancreas-specific *Zip5*-knockout (***Zip5 ^Panc KO^***) were given an I.P. injection of zinc sulfate (6.25 mg zinc/kg body weight) and 24 h later pancreata were harvested and paraffin sections were prepared, stained with hematoxylin and eosin and evaluated blind by a pathologist. Each row represents an individual animal.

**Figure 6:**
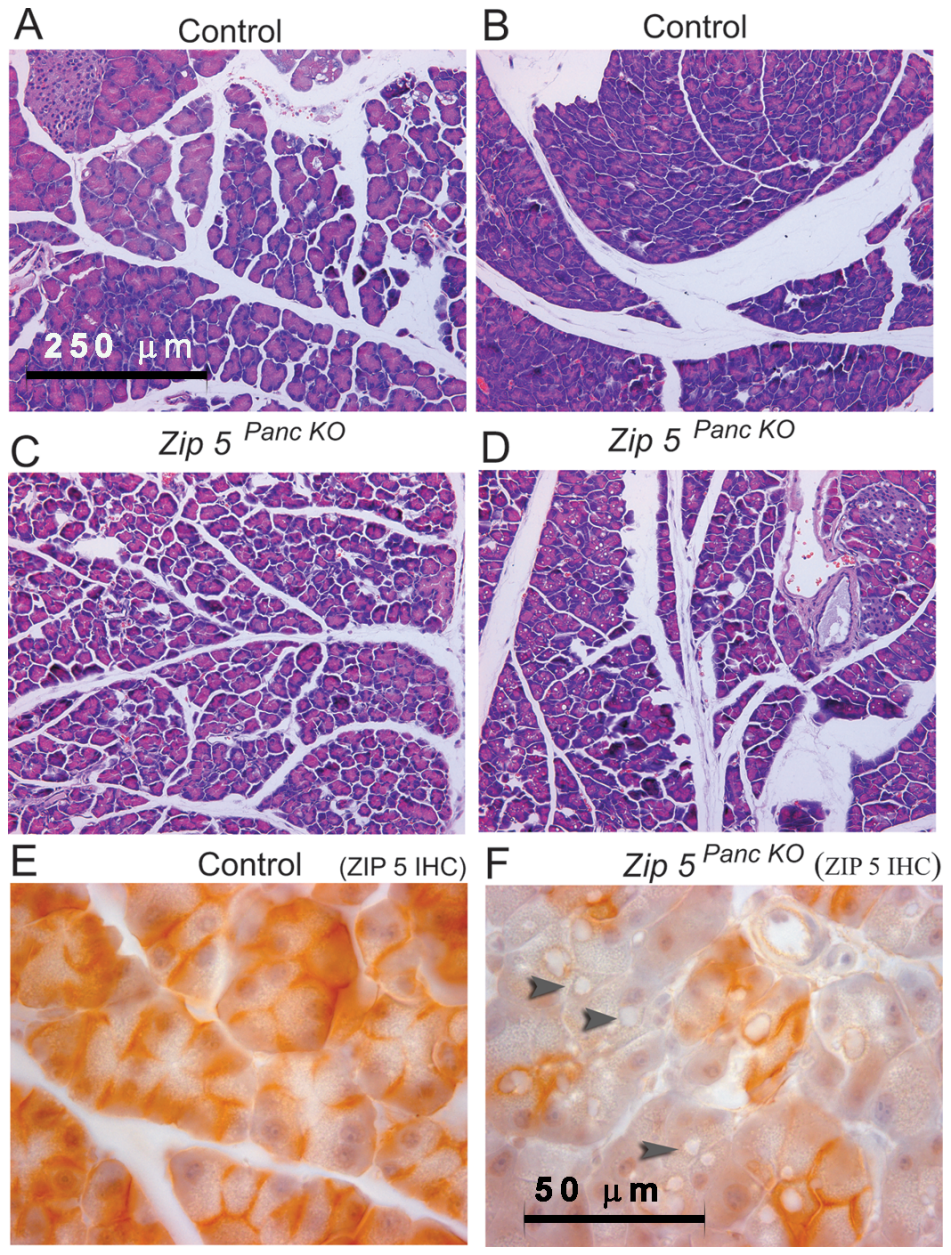
Knockdown of the pancreas acinar cell *Zip5* gene sensitizes mice to zinc-induced acute pancreatitis. Two weeks after the last tamoxifen injection, control (Control) littermates and pancreas-specific *Zip5*-knockout (*Zip5 ^Panc KO^*) (n = 5) were given an I.P. injection of zinc sulfate (12.5 mg zinc/kg body weight) and 48 hr later pancreata were harvested and paraffin sections were prepared and stained with hematoxylin and eosin (panels A–D) or stained for ZIP5 using immunohistochemistry (panels E and F). Panels A and B represent control mice whereas panels C and D represent ZIP5- knockout mice (*Zip5 ^Panc KO^*). Dark brown deposits on the basolateral surfaces of acinar cells indicate positive staining. Black arrowheads in panel F demarcate large cytoplasmic vaculoles found in acinar cells of *Zip5 ^Panc KO^* mice in response to zinc. Panels A–D are photographed at 200× magnification and panels E and F are photographed at 1000× magnification.

**Figure 7:**
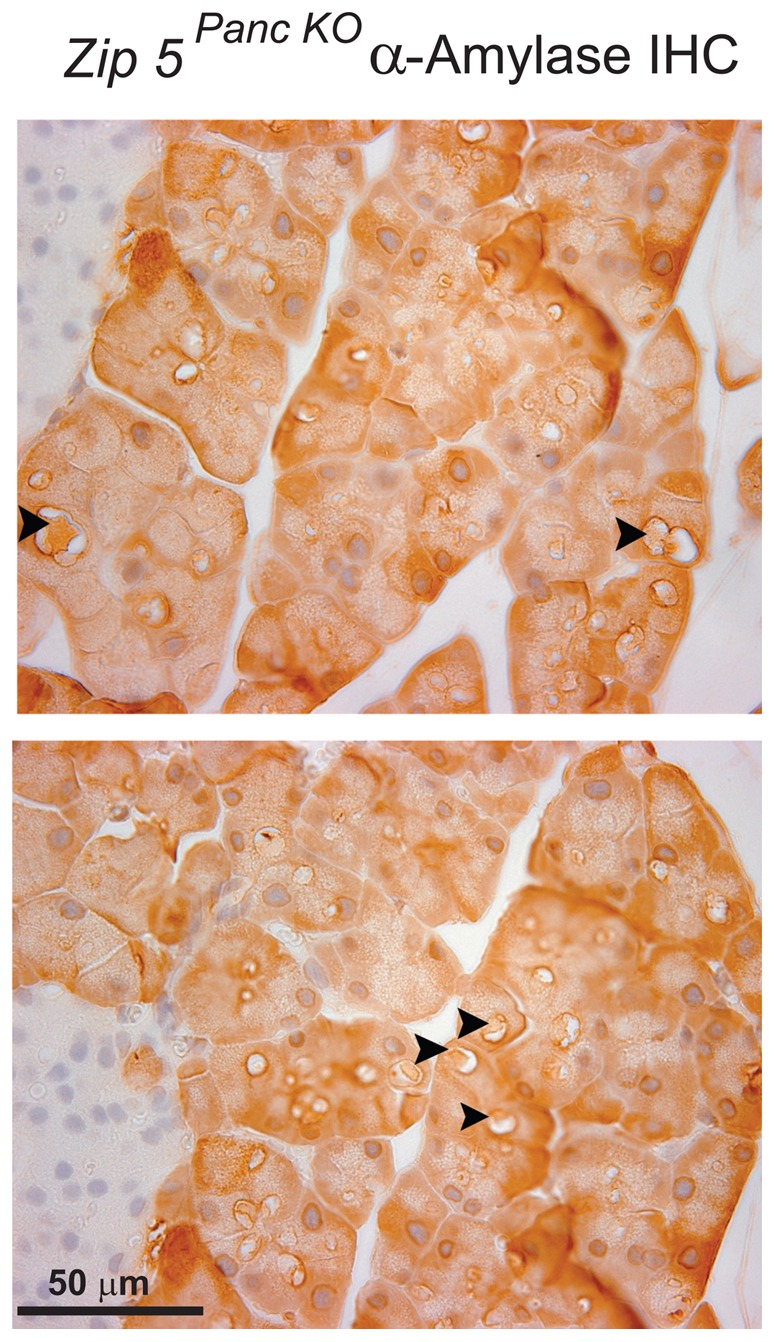
Detection of α-amylase in zinc-induced large cytoplasmic vaculoles in acinar cells of *Zip5 ^Panc KO^*mice. Two weeks after the last tamoxifen injection pancreas-specific *Zip5*-knockout (***Zip5 ^Panc KO^***) mice were given an I.P. injection of zinc sulfate (12.5 mg zinc/kg body weight) and 24 hr later pancreata were harvested and paraffin sections were prepared and stained for α-amylase using immunohistochemistry. Sections from two different mice are shown. Dark brown deposits in acinar cells indicate positive staining. **Black arrowheads** demarcate large cytoplasmic vacuoles containing α-amylase. Sections were photographed at 630× magnification. **I**; indicates an Islet of Langerhans.

To further probe this finding, mice were injected with a larger dosage of zinc (12.5 mg/kg) and the pancreas was examined by histology and IHC (Fig. 6 and Fig. 7; Table 2). Those mice showing signs of acute toxicity were euthanized within a few hours of the injection. Mice were killed at 24 to 48 hr after the zinc injection and showed no overt signs of toxicity. Control mice, *Zip5^Total KO^* mice and *Zip5 ^Panc KO^* mice were compared (Table 2). Histological examination of the pancreas 48 hr after the zinc injection revealed more severe atrophy of acinar cells in both types of knockout mice (pancreas or total knockout) than in control mice (Table 2). The severity of acinar cell atrophy was greatest in the total knockout mice (7/8 mice shown in Table 2 had severe atrophy) followed by the *Zip5 ^Panc KO^* mice (2/7 mice showed severe atrophy) whereas the majority of control mice exhibited mild atrophy of acinar cells (4/6 mice displayed mild atrophy) (see Fig. 6: panels A and B are control mice whereas panels C and D are *Zip5 ^Panc KO^* mice-). Histological examination of the pancreas revealed an abundance of large cytoplasmic vacuoles in many *Zip5 ^Panc KO^* mice (3/7) as noted above (Table 2; Figs. 6F and 7). Acinar cells with these large vacuoles were also abundant in the majority of *Zip5^Total KO^* mice (5/7 of the mice shown in Table 2) after zinc injection (Table 2) but were very rare in control mice. These large cytoplasmic vacuoles were generally noted only in acinar cells which lacked ZIP5 (Fig. 6F) although there were a few exceptions. Interestingly, these large vacuoles were present within 24 hr after the injection and contained α-amylase (Fig. 7) which is normally found in secretory vesicles. Formation of similar large vacuoles in acinar cells in other models of acute pancreatitis has been described recently [33]. The above results demonstrate that ZIP5 functions to protect the pancreas from zinc toxicity and suggest a function of this zinc transporter in the autophagy of secretory vesicles during acute pancreatitis.

**Table 2:** Zinc-induced pancreatic pathology in *Zip5 ^Panc KO^* mice, *Zip5 ^Total KO^* and control mice.

Genotype	Large Cytoplasmic Vacuoles	Acinar Cell Atrophy
***Zip5 ^Panc KO^***	**Yes**	**moderate > severe**
	**No**	**moderate > severe**
	**Yes**	**severe > moderate**
	**No**	**moderate > severe**
	**Yes**	**severe > moderate**
	**Yes (rare)**	**moderate > severe**
	**No**	**moderate > severe**
***Zip5 ^Total KO^***	**No**	**severe > moderate**
	**Yes**	**severe > moderate**
	**Yes**	**severe > moderate**
	**Yes**	**severe > moderate**
	**Yes**	**severe > moderate**
	**Yes**	**severe > moderate**
	**Yes (rare)**	**severe > moderate**
	**Yes**	**moderate > severe**
**Control**	**No**	**mild > moderate**
	**No**	**mild > moderate**
	**No**	**mild > moderate**
	**No**	**mild > moderate**
	**Yes (rare)**	**moderate > severe**
	**Yes (rare)**	**moderate > severe**

*Zip5* recombination in pancreatic acinar cells was induced by activation of CRE:ERT2 expressed from an *ela-Cre-ERT2* transgene and *Zip5* recombination in all cells was created using a ubiquitously expressed CRE recombinase. Two to three weeks after weaning, control (**Control**), pancreas-specific *Zip5*-knockout (***Zip5 ^Panc KO^***) and *Zip5*-total knockout mice (***Zip5 ^Total KO^***) were given an I.P. injection of zinc sulfate (12.5 mg zinc/kg body weight) and 48 h later pancreata were harvested and paraffin sections were prepared, stained with hematoxylin and eosin and evaluated for the presence of zinc-induced vacuoles in acinar cells and for acinar cell atrophy. Sections were evaluated by the authors in this table. Each row represents an individual animal.

## Discussion

The zinc transporter ZIP5 (*Slc39a5*) localizes to the baso-lateral surface when transfected into polarized cells as well as *in situ* in intestinal enterocytes and pancreatic acinar cells in mice [25], [34]. This protein is regulated by zinc and accumulates when zinc is replete but is internalized and degraded when zinc is deficient [26], [35]. These findings lead us to hypothesize that ZIP5 may function in the removal of zinc from the body. We tested this hypothesis by deleting the *Zip5* gene specifically in intestinal enterocytes or pancreatic acinar cells or in every cell in mice and then examining the effects on zinc homeostasis.

Although several members of the Slc39a family are known to be important for proper development of the embryo or neonate, only *Zip4* has been shown to be essential throughout the life of mice from the peri-implantation period to adulthood[16], [17]. In contrast, *Zip5* is a non-essential gene since mice lacking this gene reproduce normally and no overt problems with development are noted. However, mice lacking ZIP5 accumulate more zinc in the liver and fail to accumulate excess zinc in the pancreas. This suggests that zinc homeostasis is disturbed in these knockout mice. The accumulation of hepatic zinc is consistent with the possibility that the excretion of zinc from the body is impaired in the absence of ZIP5.

Zinc homeostasis in mammals is not well understood at the molecular level. However, our recent studies proved that ZIP4 (*Slc39a4*) is essential for the uptake of sufficient dietary zinc by the intestine [17]. Uptake of zinc is enhanced when dietary zinc is deficient and repressed when dietary zinc is adequate. However, when zinc is replete the majority of zinc taken up from the diet is excreted [36]–[38] and isotopic tracer studies indicate that intestinal excretion of zinc into the gut lumen plays a critical role in maintaining zinc homeostasis [37], [39]. Furthermore, a significant amount of zinc is found in pancreatic secretions which empty into the gut lumen, and this organ is thought to also play a role in zinc homeostasis [40]–[42]. When zinc is in excess, pancreatic metallothioneins expression is augmented and the organ accumulates a labile pool of zinc that is available during periods of zinc deficiency [43].

Our studies reveal that ZIP5 plays an important role in the intestinal excretion of zinc. The loss-of-function of this gene in the intestinal enterocytes is accompanied by increased accumulation of zinc in the pancreas. This strongly suggests that an increased body burden of zinc occurs in the absence of enterocyte ZIP5. We also noted that enterocyte *Zip4* mRNA is more abundant in these mice. Enterocyte *Zip4* mRNA is normally induced in response to zinc deficiency [44]. Therefore, these findings are consistent with the concept that ZIP5 functions to move zinc from the blood into the enterocyte for subsequent excretion into the intestinal lumen. In the absence of ZIP5 intestinal enterocytes may be mildly zinc deficient, which stabilizes *Zip4* mRNA, and the pancreas accumulates excess zinc that would normally be excreted via the intestine. However, it is clear that mice can adapt to the absence of intestinal ZIP5 under normal dietary conditions.

Our studies confirm that ZIP5 in pancreatic acinar cells also functions in zinc homeostasis. Deletion of this gene in these cells led to diminished steady state levels of pancreatic zinc similar to what was found in mice with total knockout of ZIP5. In addition, chronic excess dietary zinc failed to accumulate in pancreatic acinar cells which lack ZIP5. This effect did not appear to reflect a lack of acute uptake of zinc into acinar cells indicating that ZIP5 is not essential for zinc uptake by the pancreas. Instead our studies suggest that ZIP5 might be involved in the retention of zinc. A compartmental model of zinc kinetics in mice suggested that zinc loss from the pancreas could be explained in part by secretion into the plasma [45]. Therefore, ZIP5 is localized on acinar cells in a position to function in the reuptake of zinc from plasma. Many zinc transporters are expressed in the pancreas [38]. With regard to the expression of members of the *Slc39a* family, it was recently reported that ZIP14 localizes to the plasma membrane of acinar cells [46] suggesting that this transporter could play a dominant role in the acute uptake of zinc. In contrast, the expression of *Zip1* and *Zip3* genes is most active in ductal epithelia cells [47] which lead us to hypothesize that these transporters might also function in the retention of pancreatic zinc by scavenging zinc from the exocrine secretions. However, zinc released into the pancreatic ductal system which feeds into the intestine is thought to be largely associated with secretory proteins and metallothioneins [48]. It remains to be determined how zinc is rapidly taken up by acinar cells and the mechanism by which ZIP5 impacts zinc retention.

A novel and interesting finding in our studies is that acinar cell ZIP5 functions to attenuate zinc-induced pancreatitis. This is consistent with an important function of ZIP5 in pancreatic zinc homeostasis. However, the mechanism behind this protection from zinc toxicity is unclear. Our previous studies showed that metallothioneins, which are zinc-bound predominately, protect against caerulein-induced pancreatitis [49]. This suggests the possibility that the decreased zinc-content of acinar cells lacking ZIP5 predisposes them to a subsequent challenge with a toxic dosage of zinc. Previous studies have indicated that zinc deficiency exacerbates pancreatitis [50], [51]. Pancreatitis involves increased oxidative stress and inflammation [52] and in our studies inflammation was increased in mice lacking acinar cell ZIP5. Increased peri-pancreatic inflammation in these mice indicates that extra-pancreatic zinc toxicity was exacerbated. This could reflect an increased body burden of zinc, consistent with a role of pancreatic ZIP5 in the excretion of zinc. Surprisingly the acute uptake of zinc did not differ between control and *Zip5^Panc KO^* mice. Therefore, it is not simply increased cytoplasmic zinc content that dictates the toxicity of zinc in this model.

Another novel finding in our studies is that in the absence of ZIP5 many acinar cells become filled with large cytoplasmic vacuoles containing secretory protein when challenged with a toxic dosage of zinc. Impaired autophagy has been shown to mediate acinar cell vacuole formation in rodent models of acute pancreatitis [29], [33], [53]. Vaccaro [28] has termed this process “zymophagy” indicating the autophagy of zymogen granules. These vacuoles can be larger than the nucleus in some models of pancreatitis; for example in LAMP-2 deficient mice [54]and N-acetylglucosamine-1-phostransferase deficient mice (see Fig. 5 in [53]). Our results reveal that this process of zymophagy is exacerbated during zinc-induced pancreatitis in mice lacking acinar cell ZIP5. Autophagic, lysosomal and mitochondrial dysfunctions have been proposed to be keys to the pathogenesis of pancreatitis [29]. Thus, our studies quite unexpectedly suggest that ZIP5 may also have important functions in autophagy. ZIP5 undergoes internalization and degradation in response to zinc deficiency [26], [27] and can therefore be associated with components of the vesicular compartment. The intriguing possibility of a function of ZIP5 in autophagy warrants further investigation.

## Materials and Methods

### Animals

Experiments involving mice were performed in accordance with the guidelines from the National Institutes of Health for the care and use of animals and were approved by the University of Kansas Medical Center Institutional Animal Care and Use Committee (Protocol #2012-2057).

### BAC Recombineering of a floxed *Zip5* (*Slc39a5*) gene

We previously described the structure of the mouse *Zip5* (*Slc39a5*) gene in detail [25]. The final structure of the *floxed Zip5* (*Zip5^Fx^*) gene targeting vector and of the targeted chromosomal locus is shown in Fig. 1. BAC recombineering [55] using galK selection was employed to manipulate the *Zip5* gene [56]. An 8910 bp region containing the *Zip5* gene was captured from BAC ct7-575j15 and gap-repaired in a conditionally amplifiable [57] BAC-based vector (P[acman]-M-KO) [58] that allows for negative selection (*HSV-TK*) in ES cells and positive selection in bacteria (*ampicillin*). A *LoxP* site engineered to contain an *EcoRV* restriction site was inserted into a poorly conserved region in intron 4 and a *PGK-neomycin* cassette was inserted about 32 bp downstream of exon 12. A second *LoxP* site was inserted 40 bp downstream of *PGK-neomycin*. The targeting vector's structure was confirmed for the entire gene including the neomycin cassette by DNA sequencing and the ends of the captured regions were sequenced to verify that no rearrangements or mutations had occurred. In addition, the functionality of the *LoxP* sites was confirmed by transformation of the final targeting vector into bacteria (EL 350) that express Cre recombinase under control of an arabinose inducible promoter [55].

### Targeted insertion of the *Zip5^Fx^* gene in embryonic stem cells

The Transgenic and Gene-Targeting Institutional Facility at the KU Medical Center generated targeted ES cell clones and performed blastocyst injections. The *Zip5* ^Fx^ targeting vector was electroporated into E14 embryonic stem (ES) cells and colonies were screened using long range PCR with LA-Taq (TaKaRa Bio, Inc.) and primers outside the captured *Zip5* locus paired with primers within the *Zip5* gene itself (Fig. 1). Primers flanking the *LoxP* site in intron 4 were used for genotyping the targeted allele in mice (Fig. 1). Homologous recombination of the targeting vector into the endogenous locus resulted in the insertion of an *EcoRV* site in intron 4 which aided in identifying the targeted alleles. The sequences of oligonucleotides for integration screen and genotyping are shown in Table SI.

### Generation of mice with *Zip5^Fx/Fx^* alleles

Chimeric mice were generated by microinjection of two independent *Zip5^Fx/Wt^* ES cell clones into d4 C57BL/6 blastocysts, followed by transfer to pseudopregnant CD-1 foster mothers. Resulting chimeric mice were crossed with C57BL/6 females (Harlan labs). Germline transmission was confirmed by PCR from tail DNA of agouti offspring (Fig. 1). *Zip5^Fx/Wt^* mice were crossed and *Zip5^Fx/Fx^* offspring were identified by PCR amplification of the *LoxP:EcoRV* insertion in intron 4.

### Generation of mice for inducible recombination of *Zip5^Fx/Fx^* alleles in the intestinal epithelium or pancreatic acinar cells

*Zip5^Fx/Fx^* mice were crossed to create a working colony of mice homozygous for floxed *Zip5* genes. These mice were then crossed with transgenic mice bearing a tamoxifen-dependent Cre recombinase (*vil-Cre-ERT2*) expressed under the control of the *villin* promoter to allow for inducible recombination of the *Zip5* gene specifically in the intestinal epithelium [30] or they were crossed with transgenic mice bearing a tamoxifen-dependent Cre recombinase (*Ela-Cre-ERT2*) expressed under the control of the *elastase* promoter to allow for inducible recombination of the *Zip5* gene specifically in pancreatic acinar cells [31]. Offspring were backcrossed to yield mice heterozygous for *Cre-ERT2* and homozygous *Zip5^Fx/Fx^*. These mice were then crossed with *Zip5^Fx/Fx^* mice to yield 50% offspring with *Zip5^Fx/Fx^*: *Cre-ERT2* alleles and 50% with *Zip5^Fx/Fx^* alleles. The latter provided age and genetically matched controls for our experiments and were labeled as control (Con) in all the figures.

### Generation of *Zip5* (*Slc39a5*) knockout mice

*Zip5^Fx/Fx^* mice were mated with transgenic mice (strain name: B6.FVB-TgN (EIIa-Cre) C5379 Lmgd from JAX.org) which express Cre ubiquitously driven by the EIIa promoter. The extent of the knockout was monitored by genotyping DNA from tail snips. Mice with apparently complete recombination of this gene in tail DNA were crossed and offspring were genotyped to confirm that the knockout was complete. A colony of homozygous *Zip5-* knockout mice was then established. *Zip5^Fx/Fx^* mice served as the control strain for these studies.

### Tamoxifen induction of recombination

A tamoxifen stock solution was prepared and injected as described in detail previously [17], [59]. Recently weaned mice (5 to 8 days post-weaning) were injected I.P. with 100 µl (1 mg tamoxifen) daily for 5 consecutive days.

### Experimental designs

Diets were purchased from Harlan Teklad (teklad.com) and zinc levels in the diets were as follows: zinc-deficient (ZnD), <1 ppm zinc; zinc-adequate (ZnA), 50 ppm zinc. Recently weaned mice were maintained on ZnA chow and the liver, pancreas and small intestine were taken 8 to 12 days after initiation of the tissue-specific knockout or tissues were harvested from total knockout mice of the same age. To monitor zinc accumulation mice were provided with drinking water containing 250 ppm ZnSO_4_ (zinc excess: ZnE) for 8 days before harvest. Where indicated, some mice were gavaged (100 µl) with a slurry containing ZnD feed in deionized water (1g feed in 2 ml water) to which was added 30 ppm zinc or to which was added ^67^Zn (250 ppm or 500 ppm). To look at the rapid accumulation and retention of zinc, where indicated mice were injected I.P. with ^67^Zn (>95% enriched, ^67^ zinc oxide) from Trace Sciences International Corporation (isotopetrace.com). ^67^Zn stock was dissolved in 3 drops of concentrated HCl and then diluted to 5 mg/ml in distilled water. This stock was diluted in water to a final concentration of 125 µg ^67^Zn/100 µl and injected I.P. at a dosage of 6.25 mg zinc/kg body weight. Pancreas was harvested from 2 to 24 hr after injection of ^67^Zn and subjected to elemental analysis. Eight to ten mice per group were analyzed in this experiment.

To induce acute pancreatitis, mice were injected I.P. with ZnSO_4_ dissolved in acidified water (100 µl) at a dosages of 6.25 mg zinc/kg body weight or 12.5 mg zinc/kg, as indicated in Results and Figure legends. The pancreas, liver and intestine were harvested (4 to 9 mice per group) at 24 or 48 hr after the zinc injection. Sections of pancreas were scored independently by a pathologist where indicated or by a member of the laboratory. Peri-pancreatic inflammation refers to inflammation around the pancreas as judged by the infiltration of immune cells.

### Northern blot hybridization

Total RNA (3 - 6 µg), isolated using Trizol reagent (Invitrogen), was size-fractionated by agarose-formaldehyde gel electrophoresis, transferred and UV cross-linked to a Zeta Probe GT nylon membrane (BioRad). Membranes were hybridized, washed and exposed to film as described previously [17]. Riboprobes for mouse *Zip4* and *Zip5* were described previously [25]. Probes were used at 2×10^6^ cpm/ml of hybridization solution.

### Histology/IHC

The small intestine and pancreas (3 to 5 mice per group) were collected, washed with cold PBS, cut into small pieces and fixed in Bouin's fixative or 4% paraformaldehyde in PBS overnight at 4°C. Fixed tissues were embedded in paraffin and sections (1 µm) were prepared by Histo-Scientific Research Laboratories (HSRL) or the KU Medical Center histology core facility. Bouin's fixed sections were deparaffinized, rehydrated and stained with hematoxylin-eosin for examination of gross morphology by a pathologist.

Paraformaldehyde fixed sections from pancreas were processed using the Histostain Plus LAB SA Detection System (Invitrogen) according to the manufacturer's instructions. Sections were incubated with antisera against ZIP5 (1∶200), as described previously [25], [26] after antigen retrieval in citrate or sections were incubated with antisera against α-amylase (1∶300; Cell Signaling). Stained slides were counterstained briefly in Mayer's hematoxylin (Sigma) and photographed using a Leica DM 4000B microscope (Leica-microsystems) with Adobe Photoshop image capture software (Adobe).

### Elemental and essential metal determination

Elemental profiling via ICP-MS was performed at the Donald Danforth Plant Science Center Ionomics Core Facility in St. Louis, Missouri [60] and at the Purdue University Ionomics Facility, in West Lafayette, Indiana [61]. Mouse tissues (n = 3 to 5) were dried at 95°C in a vacuum oven, digested in concentrated HNO_3_ and the following elements were measured: Na, Mg, P, K, S, Ca, Fe, Co, Cu, Zn, Mn, Mg, As, Se, and Mo as described previously [12], [61]. In addition, the stable isotopes of zinc, ^67^Zn and ^66^ Zn were measured when indicated. The natural ratio of these zinc isotopes is 0.146. Tissue concentrations were determined as µg g^−1^ dry weight (ppm) of each element.

### Statistical analyses

Graphs were generated and statistical analyses performed using GraphPad Prism5 software (GraphPad Software). Statistical significance was determined using the Unpaired T-test (two-tailed) and values were considered different if P<0.05. Data are expressed as the mean ± S.E.M. *indicates P<0.05; *** indicates P<0.001; **** indicates P<0.0001

## Supporting Information

Figure S1 **Liver iron and pancreatic zinc are reduced in** ***Zip5 ^Panc KO^*** **mice.** Control (**Con**) littermates and pancreas-specific *Zip5*-knockout (***Zip5 ^Panc KO^***) mice were killed 8 days after the last tamoxifen injection. Intestine, pancreas and liver were harvested from mice fed normal chow (**ZnA**) during those 8 days and elements were quantified using ICP-MS and are expressed as ppm/dry weight of tissue. (**A**) Liver iron (n = 4 –5 mice per group). (**B**) Pancreatic zinc (n = 8 –10 mice per group) is expressed as the ratio of ^66^Zn to sulfur (**S**). This was done to normalize the values for zinc and reduce variability. Two separate groups of mice were analyzed thus there are two sets of data on this bar graph. ^66^Zn represents ∼28% of total zinc. There were no apparent changes in any of the other elements analyzed in these tissues.
(TIF)

Figure S2 ***Zip5 ^Panc KO^*** **mice appear to display reduced retention of zinc.** (**A**) Intestine-specific *Zip5*-knockout (***Zip5 ^Intest KO^***) mice and pancreas-specific *Zip5*-knockout (***Zip5 ^Panc KO^***) mice were given an oral gavage containing 250 ppm or 500 ppm ^67^Zn and the pancreas was harvested 24 hr after the gavage (n = 4 mice per group. The ratio of ^67^Zn/^66^Zn was measured by ICP-MS. The natural ratio of these stable isotopes is 0.146. (**B**) Mice were given an oral gavage containing 500 ppm ^67^Zn and the pancreas and intestine were harvested 24 hr after the gavage. None of the values shown reached statistical significance but the data suggest a trend toward reduced retention of pancreatic zinc in the *Zip5 ^Panc KO^* mice.
(TIF)

Table S1 **List of oligonucleotides used for integration screening in embryonic stem cells and genotyping of** ***Zip5*** **alleles in mice**.
(DOCX)
