# Supplementary figures and images for: The Zinc Transporter Zip5 (Slc39a5) Regulates Intestinal Zinc Excretion and Protects the Pancreas against Zinc Toxicity
Source: PLoS One. 2013 Nov 26;8(11):e82149. doi: 10.1371/journal.pone.0082149 (PMC3841122; doi:10.1371/journal.pone.0082149)

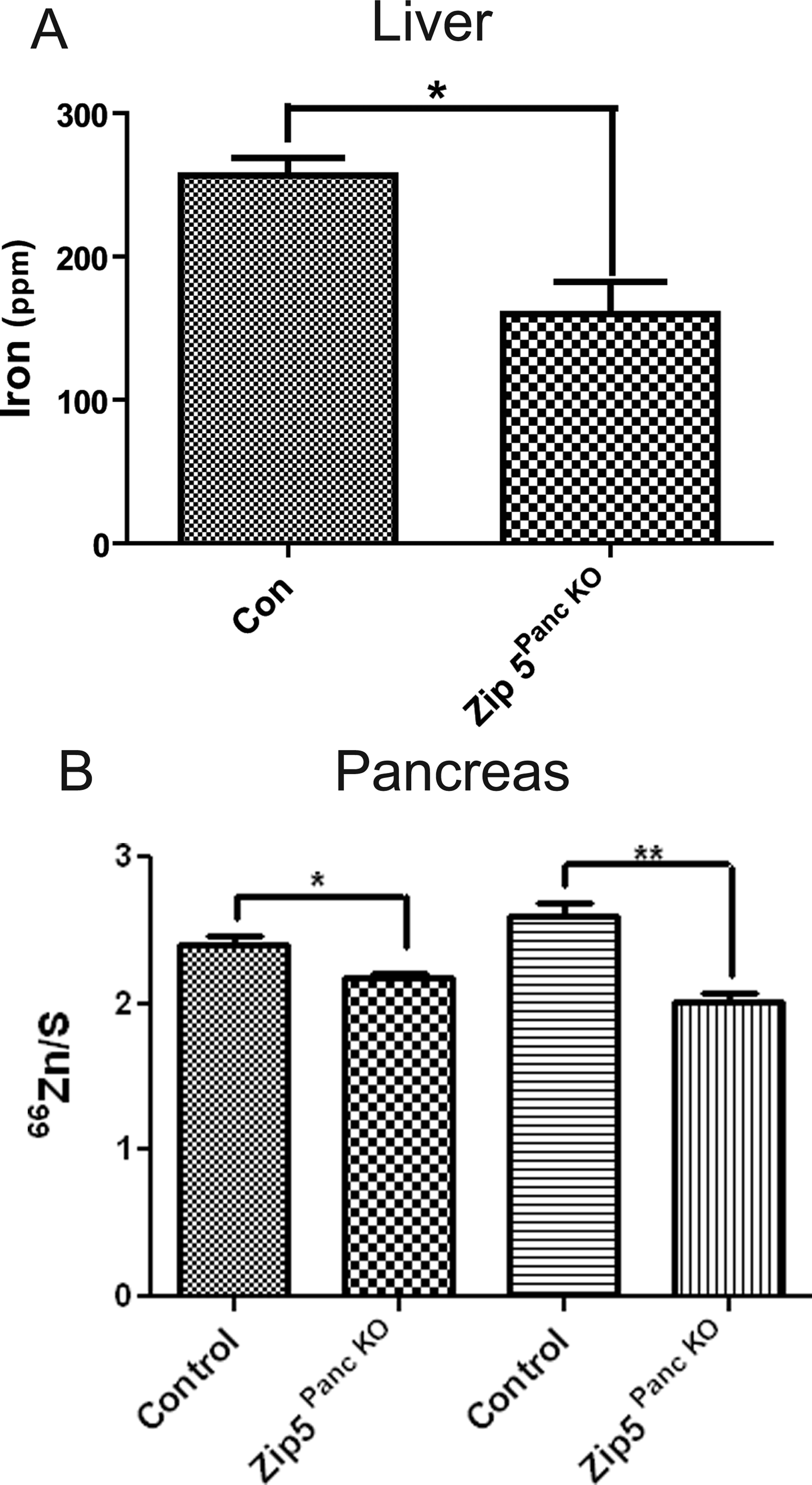

Supplement: Figure S1 — Liver iron and pancreatic zinc are reduced in Zip5 Panc KO mice. Control (Con) littermates and pancreas-specific Zip5-knockout (Zip5 Panc KO) mice were killed 8 days after the last tamoxifen injection. Intestine, pancreas and liver were harvested from mice fed normal chow (ZnA) during those 8 days and elements were quantified using ICP-MS and are expressed as ppm/dry weight of tissue. (A) Liver iron (n = 4 –5 mice per group). (B) Pancreatic zinc (n = 8 –10 mice per group) is expressed as the ratio of 66Zn to sulfur (S). This was done to normalize the values for zinc and reduce variability. Two separate groups of mice were analyzed thus there are two sets of data on this bar graph. 66Zn represents ∼28% of total zinc. There were no apparent changes in any of the other elements analyzed in these tissues. (TIF) [file pone.0082149.s001.tif]

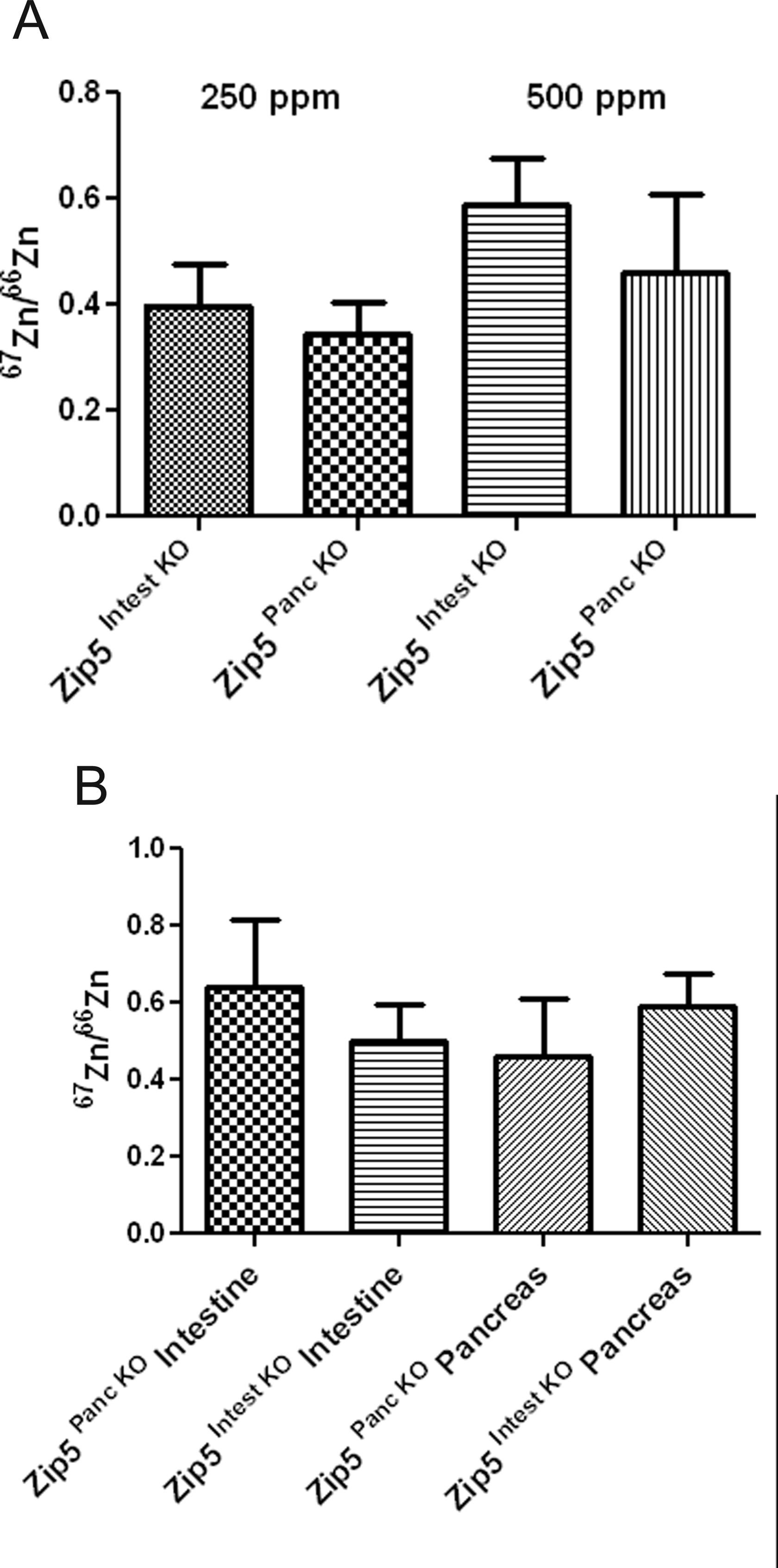

Supplement: Figure S2 — Zip5 Panc KO mice appear to display reduced retention of zinc. (A) Intestine-specific Zip5-knockout (Zip5 Intest KO) mice and pancreas-specific Zip5-knockout (Zip5 Panc KO) mice were given an oral gavage containing 250 ppm or 500 ppm 67Zn and the pancreas was harvested 24 hr after the gavage (n = 4 mice per group. The ratio of 67Zn/66Zn was measured by ICP-MS. The natural ratio of these stable isotopes is 0.146. (B) Mice were given an oral gavage containing 500 ppm 67Zn and the pancreas and intestine were harvested 24 hr after the gavage. None of the values shown reached statistical significance but the data suggest a trend toward reduced retention of pancreatic zinc in the Zip5 Panc KO mice. (TIF) [file pone.0082149.s002.tif]
