# Supplementary material for: The Zinc Transporter Zip5 (Slc39a5) Regulates Intestinal Zinc Excretion and Protects the Pancreas against Zinc Toxicity
Source: PLoS One. 2013 Nov 26;8(11):e82149. doi: 10.1371/journal.pone.0082149 (PMC3841122; doi:10.1371/journal.pone.0082149)
Supplement: Table S1 — List of oligonucleotides used for integration screening in embryonic stem cells and genotyping of Zip5 alleles in mice. (DOCX) [file pone.0082149.s003.docx]

**Table S1**  List of oligonucleotides used for integration screening in embryonic stem cells and genotyping of *Zip5* alleles in mice.

5’ long range PCR (s) tcttctacaagagcagccagtgctcctcactgc

5’ long range PCR (as) cctctgggtcagggtttggttctccaagatcc

6.22 kb; RV cuts Fx to 4.5 and 1.7 kb products.

3’ long range PCR (s) agaaccaaaccctgacccagaggatggcag

3’ long range PCR (as) acccctatccgttctgactgtaagtccagctcacc

Wt; 3.39 kb: Fx; 5.26 kb

Zip5 genotyping PCR (s) atccagggaagtatctcagggttagg

Zip5 genotyping PCR (as) catgccacctgatcaagggtc

Zip5 post CRE (as) aatggggaccttggtgcatttg

Fx; 197 bp: Wt: 157 bp: Post Cre; 275 bp
